# A Portable Triboelectric Nanogenerator Based on Dehydrated Nopal Powder for Powering Electronic Devices

**DOI:** 10.3390/s23094195

**Published:** 2023-04-22

**Authors:** Ernesto A. Elvira-Hernández, Omar I. Nava-Galindo, Elisa K. Martínez-Lara, Enrique Delgado-Alvarado, Francisco López-Huerta, Arxel De León, Carlos Gallardo-Vega, Agustín L. Herrera-May

**Affiliations:** 1 Facultad de Ingeniería Mecánica y Ciencias Navales, Universidad Veracruzana, Calzada Ruiz Cortines 455, Boca del Río 94294, Veracruz, Mexico; aelvirah@hotmail.com; 2 Campus Torrente, Universidad Cristóbal Colón, Av. Salvador Díaz Mirón 2602, Veracruz 91910, Veracruz, Mexico; 3 Departamento de Ingeniería Mecánica, DICIS, Universidad de Guanajuato, Salamanca 36885, Guanajuato, Mexico; iem.nava@gmail.com; 4 Micro and Nanotechnology Research Center, Universidad Veracruzana, Calzada Ruiz Cortines 455, Boca del Río 94294, Veracruz, Mexico; karina.24_lara@outlook.es (E.K.M.-L.); endelgado@uv.mx (E.D.-A.); 5 Facultad de Ciencias Químicas, Universidad Veracruzana, Calzada Ruiz Cortines 455, Boca del Río 94294, Veracruz, Mexico; 6 Facultad de Ingeniería Eléctrica y Electrónica, Universidad Veracruzana, Calzada Ruiz Cortines 455, Boca del Río 94294, Veracruz, Mexico; frlopez@uv.mx; 7 CONACYT-Centro de Investigación en Química Aplicada, Boulevard Enrique Reyna 140, Saltillo 25294, Coahuila, Mexico; arxel.deleon@ciqa.edu.mx; 8 Centro de Investigación en Química Aplicada, Boulevard Enrique Reyna 140, Saltillo 25294, Coahuila, Mexico; carlos.gallardo@ciqa.edu.mx; 9 Facultad de Ingeniería de la Construcción y el Hábitat, Universidad Veracruzana, Calzada Ruíz Cortines 455, Boca del Río 94294, Veracruz, Mexico

**Keywords:** dehydrated nopal powder, energy harvesting, green energy, triboelectric nanogenerator, vibrations

## Abstract

Triboelectric nanogenerators (TENGs) based on organic materials can harvest green energy to convert it into electrical energy. These nanogenerators could be used for Internet-of-Things (IoT) devices, substituting solid-state chemical batteries that have toxic materials and limited-service time. Herein, we develop a portable triboelectric nanogenerator based on dehydrated nopal powder (NOP-TENG) as novel triboelectric material. In addition, this nanogenerator uses a polyimide film tape adhered to two copper-coated Bakelite plates. The NOP-TENG generates a power density of 2309.98 μW·m^−2^ with a load resistance of 76.89 MΩ by applying a hand force on its outer surface. Furthermore, the nanogenerator shows a power density of 556.72 μW·m^−2^ with a load resistance of 76.89 MΩ and under 4*g* acceleration at 15 Hz. The output voltage of the NOP-TENG depicts a stable output performance even after 27,000 operation cycles. This nanogenerator can light eighteen green commercial LEDs and power a digital calculator. The proposed NOP-TENG has a simple structure, easy manufacturing process, stable electric behavior, and cost-effective output performance. This portable nanogenerator may power electronic devices using different vibration energy sources.

## 1. Introduction

Due to the growth of the Internet of Things (IoT), the number of devices will increase by over 200 billion by 2025 [1]. These devices will require low-cost and environment-friendly power sources. Conventional electrochemical batteries have been used to power devices, although these batteries have limited-service time and must be constantly recharged or replaced [2,3,4]. In addition, the electrochemical batteries contain toxic materials that could cause environmental pollution. For this reason, green energy sources are required to power future IoT devices, such as wind energy, solar radiation, thermal energy, raindrop, movement of the human body, and mechanical vibrations [5,6,7,8,9,10]. These green energy sources could use supercapacitors to store their electrical energy. These supercapacitors have the advantage of supporting more electrical charge and discharge cycles than conventional batteries. In addition, these devices can be adapted to small smart embedded systems that require reliable and maintenance-free storage systems [11].

The kinetic energy of the vibrations in the environment can be converted into electrical energy using triboelectric nanogenerators (TENGs) [12]. The working principle of the TENGs is based on triboelectrification contact and electrostatic induction [12]. The triboelectric nanogenerators have advantages such as simple structural configurations, a wide range of triboelectric materials, an easy operating mechanism, a simple fabrication process, a high output performance, and non-complex signal processing [13,14,15]. The output power of TENGs depends on charge densities and dimensions of selected triboelectric materials and the operating mode frequency [16,17,18]. Most TENGs have only employed inorganic materials as triboelectric materials. Recently, researchers [19,20,21,22,23,24,25,26,27,28,29,30,31] have reported novel triboelectric nanogenerators based on organic triboelectric materials. In addition, triboelectric nanogenerators based on organic material powder have registered good output performance [19,26,31]. The nanogenerators with triboelectric layers composed of organic material powder can be deposited on electrodes with a large surface area. This is an advantage compared to organic triboelectric layers integrated by dry leaves that have a limited surface area. Furthermore, the triboelectric layers formed by organic material powder have better mechanical strength in comparison to fragile dry leaves [32]. A potential application of TENGs based on organic triboelectric materials is smart agriculture. This smart agriculture needs IoT self-powered sensors based on biodegradable or recycled materials to decrease the damage to soil and crops [33,34]. Thus, these IoT self-powered sensors could help to increase crop yields and minimize costs.

By considering organic materials, Shaukat et al. [19] fabricated a TENG using sunflower husk powder as an active triboelectric layer. This nanogenerator used polyethylene terephthalate (PET) as the opposite inorganic triboelectric film and powered devices such as calculators and stopwatches. Nevertheless, this TENG did not include an adjustable portable frame structure for uniform contact between both triboelectric layers. Xia et al. [35] developed a TENG with waste tea leaves and reused aluminum foil bags. This TENG incorporated a power management circuit for low-power office devices. However, more studies on the durability and wear of triboelectric film are required. Tomato skin is another organic material used in hybrid nanogenerators [36]. Saqib et al. [36] designed a hybrid nanogenerator that takes advantage of tomato skin’s triboelectric and piezoelectric properties, generating instantaneous power of 5400 µW, lighting 141 commercial LEDs, and powering a low-power stopwatch. On the other hand, Feng et al. [32] proposed nanogenerators composed of plant leaves, leaf powder, and living plants. Their leaf powder-based nanogenerator can power a digital clock and light 868 LEDs. Although, stability and wear tests of the organic triboelectric material of this nanogenerator must be considered.

Nopal (opuntia ficus-indica) is a cactus that grows in semi-arid and arid environments in Mexico, South Africa, Latin America, and Mediterranean countries [37,38]. Herein, we propose a portable triboelectric nanogenerator (NOP-TENG) based on dehydrated nopal powder and polyimide film tape. The proposed nanogenerator has a compact structure that can facilitate its use and transportation for green energy harvesting from different vibration sources. This nanogenerator has advantages such as a simple and portable frame structure that allows the parallel contact of the triboelectric films, an easy manufacturing process, stable electric behavior, and cost-effective output performance. The stable output performance of the nanogenerator is suitable to power future IoT electronic devices. In addition, the use of organic or recycled materials may contribute to the circular economy by decreasing waste and environmental pollution. Dehydrated nopal powder can be adhered to some recycled material surfaces to build future low-cost nanogenerators. Moreover, this portable nanogenerator can be adapted to different surfaces under random vibrations to convert the vibration energy into electrical energy. Furthermore, the dehydrated nopal powder could adhere to electrodes with variable surface shapes.

## 2. Materials and Methods

### 2.1. Materials

Our nanogenerator used dehydrated nopal powder and polyimide film tape as triboelectric materials. These materials were adhered onto two copper-coated bakelite electrode plates (52.2 mm × 52.2 mm × 1.5 mm). The dehydrated nopal powder was obtained from Natura Bio Foods (NBF) (Zapopan, Mexico) [39,40]. This nopal powder was extracted from nopal-dehydrated leaves dropped in Mexico [39]. On the other hand, polyimide film tape (50 mm width and 60 µm thickness) was bought from Steren (Mexico City, Mexico) [41].

### 2.2. Working Mechanism

**Figure 1:**
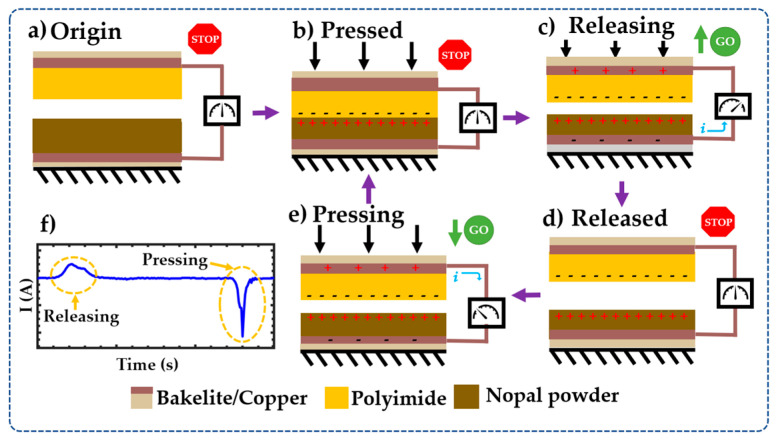
(**a**–**e**) Schematic view of the working mechanism of the NOP-TENG and (**f**) behavior of the current during an operation cycle of the NOP-TENG. In this figure, the green arrows indicate the direction of movement of the top plate of the NOP-TENG.

The working mechanism of NOP-TENG uses the vertical contact-separation mode (Figure 1), which is based on the coupling effect of contact electrification and electrostatic induction. In the original state (rest), no charges are generated or induced and there is no potential difference between the top and bottom electrodes (Figure 1a). When an external force is applied to the TENG top plate (copper-coated bakelite/polyimide film tape), it moves and contacts the TENG bottom plate (dehydrated nopal power/copper-coated bakelite). Once the two triboelectric materials are in physical contact, electrostatic charges are accumulated on the contact surfaces due to the contact electrification effect (Figure 1b). In this condition, the polyimide and nopal powder surfaces are negatively and positively charged, respectively. When the external force is removed, charges opposite to those on the surfaces of the triboelectric materials are generated in the top and bottom electrodes (Figure 1c). This charge difference produces an electrical potential drop between both electrodes, which creates a current flow through load resistance that connects the electrodes. The current flow stops when both plates reach their maximum separation distance (Figure 1d). The application of an external force again (pressing) on the top plate decreases its separation distance from the bottom plate, causing a potential drop between both electrodes and a current flow back (Figure 1e). A periodic contact and separation between the two triboelectric plates drive the current to flow back and forth between both electrodes, allowing an alternating current (AC) output signal in the external circuit (Figure 1f).

### 2.3. NOP-TENG Fabrication

**Figure 2:**
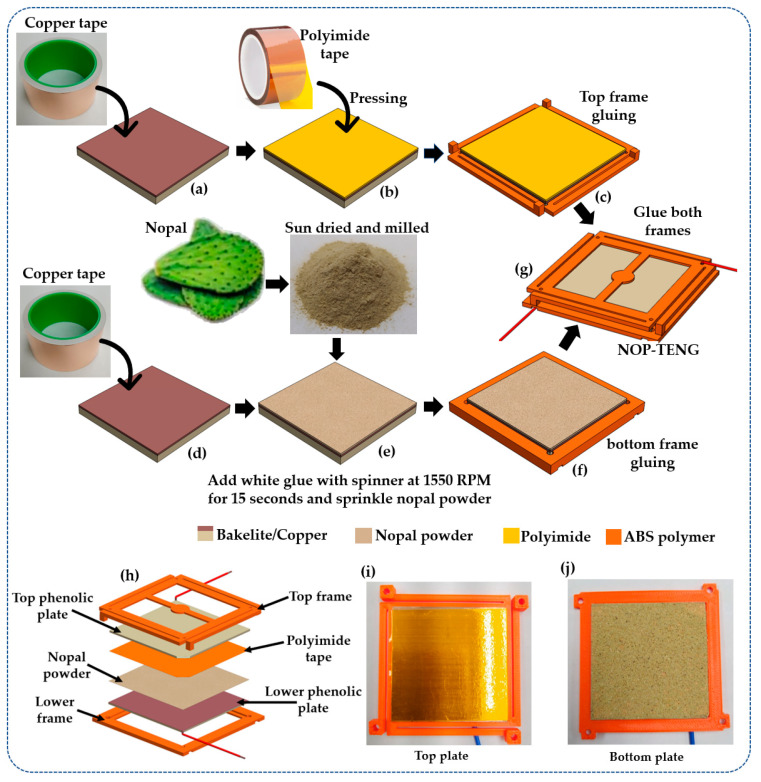
Schematic view of the NOP-TENG manufacturing process. This process begins by adhering polyimide film tape and dehydrated nopal powder on the surfaces of (**a**,**b**) top and (**d**,**e**) bottom plates. Next, both (**c**,**f**) plates are attached to 3D printing frames to form the (**g**) NOP-TENG structure. (**h**) Exploded view of the different components of the NOP-TENG. Photographs of the triboelectric materials adhered to the (**i**) top plate and (**j**) bottom plate of the nanogenerator.

In the fabrication process of the NOP-TENG, the two bakelite plates have their top surface covered with copper (Figure 2a,d). In addition, copper tape (62.2 mm × 52.2 mm × 66 μm) [32] was adhered on the top surface of each bakelite plate. This copper tape is 10.2 mm larger than the bakelite plate’s length. This copper’s additional length was adhered to the thickness and underside of the bakelite plate. Thus, the underside of the plate has 8.5 mm of copper tape that is used to solder one tinned copper wire (0.644 mm diameter). First, a polyimide film adhesive tape was attached to the copper electrode of the top plate (Figure 2b). In addition, the dehydrated nopal powder was adhered to the copper electrode of the bottom plate (Figure 2e). In order to adhere the nopal powder on the bakelite plate surface, white glue was deposited on the bottom-plate surface using the spin-coating technique at 1550 RPM for 15 s. Afterward, nopal powder was spread over the glue-coating until it was completely covered. Next, the nopal powder adhered to the glue-coating is dried at room temperature for 24 h. The residues of nopal powder were removed using compressed air. Later, tinned copper wire was soldered to the electrodes of the bakelite plates. Both plates were assembled on a flexible framework manufactured with 3D printing (Figure 2c,f), obtaining the NOP-TENG structure (Figure 2g). Figure 2h depicts the exploded view of the different components of the nanogenerator. The triboelectric materials of the NOP-TENG are shown in Figure 2i,j. This nanogenerator used polyimide film adhesive tape as the electronegative triboelectric layer and dehydrated nopal powder as the electropositive triboelectric layer. The separation distance between the bio-organic triboelectric and inorganic triboelectric layers is 1 mm.

### 2.4. Setup

**Figure 3:**
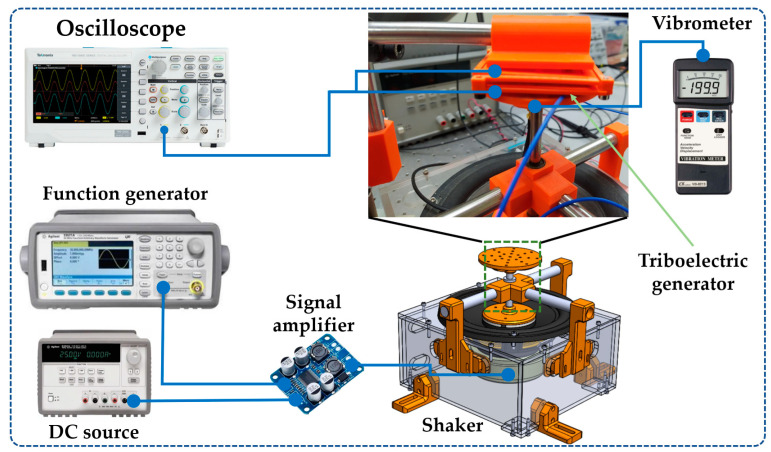
The setup used to measure the output performance of the NOP-TENG.

Figure 3 illustrates the setup used to characterize the output performance of the proposed nanogenerator. The NOP-TENG was mounted on a shaker that can generate vibrations with controlled amplitude and frequency. These vibrations were controlled by a function generator (Agilent 335000B series, Santa Clara, CA, USA), a signal amplifier (Texas instruments TPA3118, Dallas, TX, USA), and a direct current source (Agilent E3631A). The vibration accelerations were measured using a vibrometer (LUTRON VB-8213, Coopersburg, PA, USA). A digital oscilloscope (TEKTRONIX TBS1052C, Beaverton, OR, USA) was connected to two copper electrodes of the NOP-TENG using a 100 MΩ impedance oscilloscope probe. The output performance of the NOP-TENG under different vibration amplitudes at 15 Hz was measured with the oscilloscope. 

## 3. Results and Discussion

### 3.1. SEM, AFM, and EDS Characterization

**Figure 4:**
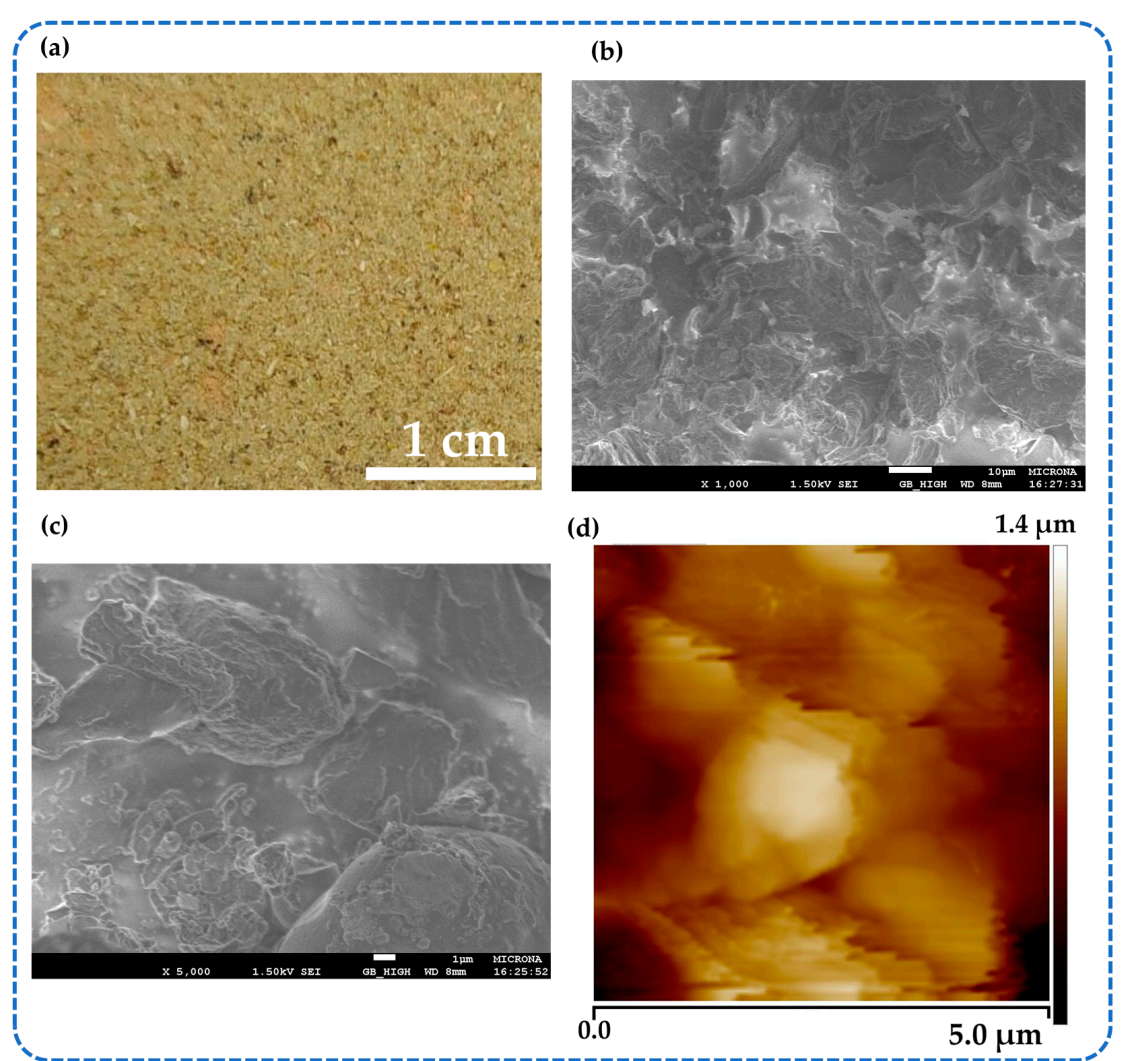
(**a**) Microphotograph of the surface of dehydrated nopal powder adhered to a copper-coated bakelite plate. SEM images (**b**) at ×1000 and (**c**) ×5000 magnification of the topological distribution of a nopal powder sample and (**d**) morphological image using AFM from the nopal powder film.

A nopal powder sample (Figure 4a) was used to study its topological distribution at the microscopic scale. Figure 4b depicts the topological distribution of the nopal powder sample using scanning electron microscopy (SEM) at ×1000 magnification with a scaling of 10 µm. Figure 4c shows an SEM image at ×5000 magnification with a scaling of 1 µm of the nopal powder sample. In these micrographs, the nopal powder film has a high roughness since it is composed of microparticles. Figure 4d depicts the morphological characteristics of the nopal powder using an atomic force microscopy (AFM) tapping image.

**Figure 5:**
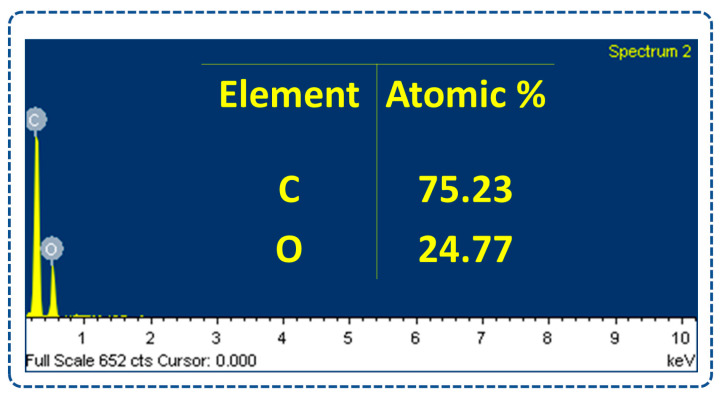
EDS results of elemental composition with atomic percentages of the nopal powder sample.

Furthermore, Figure 5 shows the energy dispersive X-ray spectroscopy (EDS) of the nopal powder sample. This EDS test detected C and O with atomic percentages of 75.23% and 24.77%, respectively. The abundant amount of oxygen present in the nopal powder confirms its electropositive nature.

### 3.2. Output Performance

**Figure 6:**
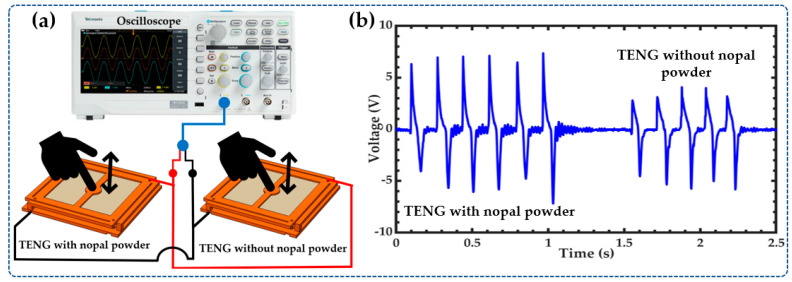
(**a**) Setup and (**b**) results of maximum *V_p-p_* of the TENGs with NOP and without NOP.

To study the triboelectric effect of the dehydrated nopal powder (NOP), the output voltages of a TENG without NOP and another with NOP were measured. For both TENGs, a polyimide film tape adheres to the copper electrode of the top plate. The first TENG included dehydrated nopal powder adhered on the surface of the bottom plate, and the second TENG had no NOP but only glue-coating on the surface of the copper electrode of the bottom plate. This glue-coating was deposited using a spin coating at 1550 RPM for 15 s. The two TENGs were electrically connected in parallel to an oscilloscope (Figure 6a), and an external force on the top plate of each TENG was manually applied via finger actuation. Figure 6b compares the open-circuit voltages produced by both nanogenerators. The first TENG generates a maximum peak-to-peak open-circuit voltage (*V_p-p_*) of 14.56 V. Instead, the second TENG has a maximum *V_p-p_* of 9.92 V. Based on these results, the maximum *V_p-p_* of the TENG with NOP increases approximately by 46.7% compared to the maximum *V_p-p_* of the TENG without NOP. Thus, the dehydrated nopal powder acts as a triboelectric material that can increase the output performance of the proposed TENG. The output performance of both TENGs is shown in Video S1 of the Supplementary Material.

**Figure 7:**
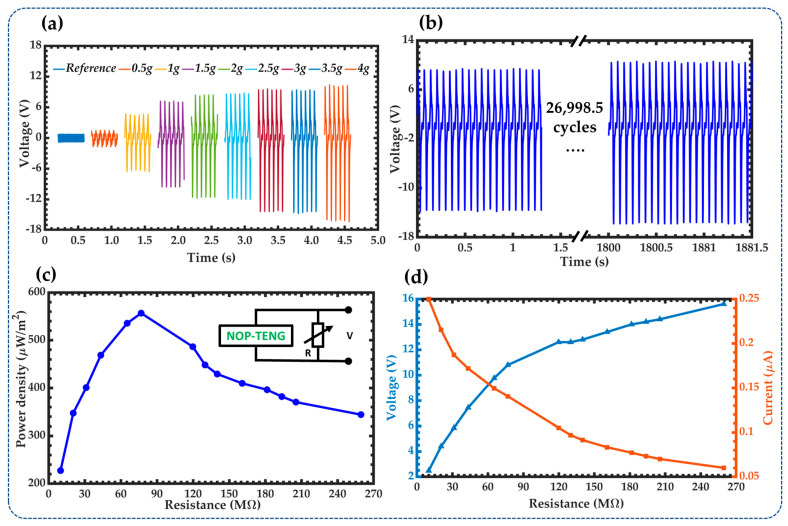
The electrical output performance of the NOP-TENG. (**a**) The open-circuit voltage of NOP-TENG under different accelerations at a frequency of 15 Hz (**b**). Stability test of the open-circuit voltage of NOP-TENG during 27,000 operating cycles (**c**). The output power density of the NOP-TENG at various load resistance from 9.92 MΩ to 259.45 MΩ and under acceleration of 4*g* at 15 Hz. (**d**). Output voltage and current of the nanogenerator at various load resistances.

The output performance of the NOP-TENG was measured using the setup of Figure 3 and by considering eight different accelerations (0.5*g*, 1*g*, 1.5*g*, 2*g*, 2.5*g*, 3*g*, 3.5*g*, and 4*g*) at the frequency of 15 Hz. The maximum peak-to-peak open-circuit voltages (Figure 7a) of the NOP-TENG are 3.16 V, 11.2 V, 16.8 V, 20 V, 20.6 V, 23.8 V, 24.2 V, and 26.6 V for the accelerations of 0.5*g*, 1*g*, 1.5*g*, 2*g*, 2.5*g*, 3*g*, 3.5*g*, and 4*g*, respectively. To study the stability of the output performance of the nanogenerator, we measure its output open-circuit voltage under acceleration of 4g at 15 Hz during 27,000 operating cycles (Figure 7b). According to these results, the *V_p-p_* the NOP-TENG has a steady state with a slowly increased trend. This increase may be attributed to the contact electrification effect between both triboelectric materials of the NOP-TENG. Additionally, the output power density of the NOP-TENG as a function of load resistance (RL) is shown in Figure 7c. For this test, load resistances with values from 9.92 MΩ to 259.45 MΩ were connected between the two electrodes of the nanogenerator under an acceleration of 4g at 15 Hz. The output power density achieves a maximum value of 556.72 μW·m^−2^ with a load resistance of 76.89 MΩ. Figure 7d illustrates the relationship between the output voltage and current of the nanogenerator with the load resistance. Figure 7d shows that the output voltage rapidly increases between 9.92 MΩ and 76.89 MΩ. However, the output current registers an inverse trend, where the current decreases with the increase in the load resistance value.

**Figure 8:**
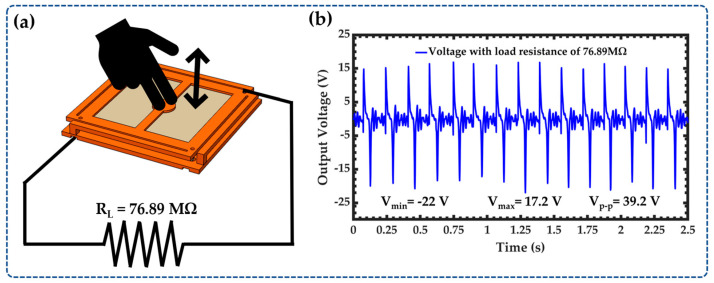
The output voltage of the NOP-TENG by applying force with the hand. (**a**) Electrical connection diagram of the load resistance to the NOP-TENG and (**b**) voltage of the NOP-TENG measured with the oscilloscope when a load resistance of 76.89 MΩ is connected between its upper and lower electrodes.

The output performance of the NOP-TENG was tested by applying a hand force on its outer surface. Based on this test, the nanogenerator has a power density of 2309.98 μW·m^−2^ with a load resistance of 76.89 MΩ. Figure 8 depicts the electrical connection diagram of the nanogenerator with the load resistance and the results of the closed-circuit voltage. Video S2 of the Supplementary Material shows the test of the output performance of the nanogenerator by applying force with the hand.

**Figure 9:**
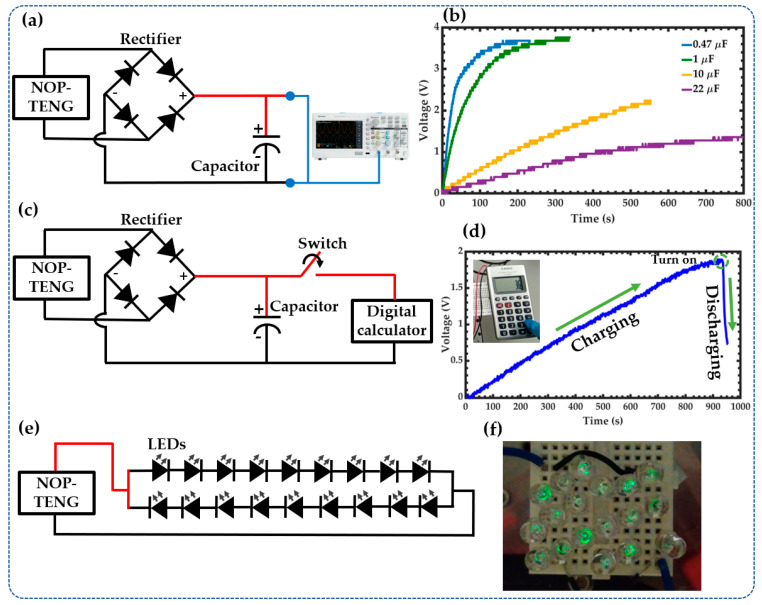
Applications of the NOP-TENG. (**a**) Schematic diagram of the electrical circuit of the NOP-TENG with a diode bridge and capacitor. (**b**) The charging curves of different capacitors. (**c**) The electrical circuit of the NOP-TENG with a diode bridge, capacitor of 22 μF, and digital calculator. (**d**) The energy scavenged from NOP-TENG is used to power a digital calculator. (**e**) The electrical circuit of the NOP-TENG is connected to 18 commercial green LEDs. (**f**) NOP-TENG can light 18 commercial green LEDs.

The NOP-TENG generates an AC signal that can be rectified with a diode bridge and stored in a capacitor. For this test, the output of the NOP-TENG was connected to a commercial diode bridge. In addition, electrolytic capacitors were connected to a diode bridge (Figure 9a). Afterward, the nanogenerator was excited with an acceleration of 2*g* at 15 Hz. The rectified signal was employed to charge four different capacitors. Figure 9b depicts the charging performance of four capacitors (0.47 μF, 1 μF, 10 μF, and 22 μF). The 0.47 μF and 1 μF capacitors charged quickly up to 3.6 V for a period of 250 s. 

Finally, we applied the NOP-TENG to power small electronic devices. For this, a 22 μF capacitor was charged for a period of 900 s with an acceleration of 4*g* at 15 Hz (Figure 9c). Figure 9d shows the voltage stored in the 22 μF capacitor until it reaches 1.9 V and its discharge voltage when the digital calculator is turned on. Video S3 of the Supplementary Material depicts the operation of the digital calculator powered by the NOP-TENG. Additionally, the NOP-TENG was used to light 18 commercial green LEDs, as shown in Figure 9e,f. Video S4 of the Supplementary Material shows the NOP-TENG lighting the 18 LEDs.

The proposed nanogenerator has a portable structure for its easy application by green energy harvesting from different vibration sources. This nanogenerator can be employed on different environment surfaces under random vibrations. For instance, an array of portable nanogenerators can be implemented on different sources of mechanical vibrations such as bridges, buildings, machine tools, and automobiles. In addition, rectifier circuits can be used to convert the AC output signal of the nanogenerators into DC signals. These DC signals can be stored in a capacitors array. Thus, these capacitors can be charged and discharged to power other electronic device types such as pressure, temperature, and humidity sensors. 

The proposed nanogenerator has a low-cost and simple manufacturing process as well as a stable output performance to power future IoT electronic devices. Moreover, the use of organic or recycled materials is suitable for the circular economy by decreasing waste and environmental pollution. For instance, dehydrated nopal powder can be adhered to some recycled material surfaces to develop cost-effective triboelectric nanogenerators.

**Figure 10:**
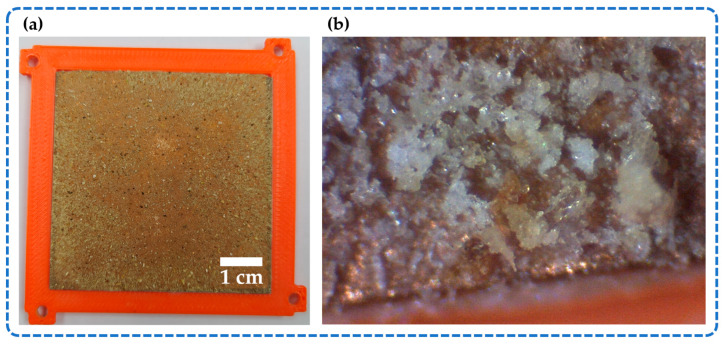
(**a**) Dehydrated nopal powder film of the bottom plate of the NOP-TENG after the stability test and (**b**) Optical microscope image (4× magnification) of a surface area of the dehydrated nopal powder film with wear effect.

Due to the friction and impact between both triboelectric layers of the NOP-TENG, the dehydrated nopal powder film suffered wear as shown in Figure 10a,b. Figure 10a shows the bottom plate of the NOP-TENG after the stability test. Furthermore, an optical microscope image at 4× magnification of the bottom plate of our nanogenerator is illustrated in Figure 10b. We observed that some particles of the nopal powder film have been detached as shown in Figure 10b. To decrease the wear and impact effect on the organic triboelectric material, the nopal powder particles must strongly adhere to the copper electrode.

### 3.3. Advantages and Weaknesses

Table 1 depicts the main advantages and drawbacks of the NOP-TENG compared to other TENGs composed of organic and inorganic triboelectric materials. Our nanogenerator has advantages such as portable and simple structural configuration, low-cost manufacturing, and stable performance that allows its application on different surfaces under mechanical vibrations with variable amplitudes. Furthermore, the output performance of the nanogenerator can be improved by increasing the pressure on the surface of the nanogenerator. In addition, this output performance could be increased to optimize the surface area, thickness, and gap of the triboelectric layers. On the other hand, more investigations about the tapping speed on the nanogenerator surface are required to study its influence on the output power density of the nanogenerator.

The proposed nanogenerator has a biodegradable triboelectric material that suffers wear due to friction. This wear of the dehydrated nopal powder is a weakness of our nanogenerator. To increase the lifetime of the organic triboelectric film, the dehydrated nopal powder must have strong adhesion to the copper electrode, obtaining a better homogenous surface layer. Nevertheless, more investigations on the lifetime of the proposed organic triboelectric material are required by considering the effect of the size and thickness of the dehydrated nopal powder as well as the influence of the environmental humidity.

Our nanogenerator needs a rectifier circuit to convert its AC output signal into a DC signal. In addition, this DC signal can be stored by employing capacitors to power electronic devices. An important challenge of triboelectric nanogenerators is their coupling with cost-effective energy storage systems. Energy management strategies of the proposed nanogenerator can be considered by adding electrical interfaces of high efficiency and minimum power consumption [42,43]. Another key challenge of the TENGs is related to their performance reliability. For this, future research works will require more reliability tests that include the durability and failure mechanisms of the NOP-TENG, incorporating the influence of high variations of temperature as well as the wear and mechanical impact strength.

## 4. Conclusions

This paper reported a novel triboelectric nanogenerator based on dehydrated nopal powder and polyimide film tape to harvest green energy from vibrations. This nanogenerator was developed using a simple portable structure with a low-cost manufacturing process. The output performance of the nanogenerator was measured by considering various accelerations and load resistances. The output voltage of the nanogenerator showed a stable behavior during a period of 27,000 operation cycles. The output power density of the nanogenerator reaches a maximum value of 556.72 μW·m^−^^2^ with a load resistance of 76.89 MΩ under 2 g acceleration at 15 Hz. This nanogenerator depicted a cost-effective output performance with the ability to power a digital calculator and light 18 LEDs.

## Figures and Tables

**Table 1:** Comparison of operating parameters of various TENGs based on organic and inorganic triboelectric materials.

Organic Triboelectric Material	Opposite Triboelectric Film	Triboelectric Layer Area	Output Power Density	Open-circuit Voltage	Advantages	Weaknesses	Ref.
Sunflower husk powder	PET	5 × 5 cm^2^	480 mW·m^−2^ at *R_L_* of 3 MΩ	488 V	Cost-effective and eco-friendly TENG	Wear of film by friction	[19]
Egg shell membrane	PTFE	6 × 8 cm^2^	250 mW·m^−2^ at *R_L_* of 2.3 MΩ	-- *	Low-cost fabrication and simple structure	Fragile structure	[20]
Fish gelatin film	PFTE/Polydimethylsiloxane (PDMS) composite film	5 × 5 cm^2^	458 mW·m^−2^ at *R_L_* of 10 MΩ	130 V	Economical, simple, and environmental-friendly structure	Wear of film by friction	[21]
Grass carp fish bladder film	Fluorinated ethylene propylene (FEP) film	4 × 4 cm^2^	243.75 mW·m^−2^ at *R_L_* of 10 MΩ	106 V	Flexible, biodegradable, and durable TENG structure	Depend on the availability of grass carp fish bladder	[22]
Rice paper	Polyvinyl chloride (PVC)	3 × 3 cm^2^	376.4 mW·m^−2^ at *R_L_* of 70 MΩ	244 V	Biodegradable, stable, and recyclable TENG structure	Wear of film by friction	[23]
Pistachio	PTFE	4.5 × 4.5 cm^2^	4161.4 mW·m^−2^ at *R_L_* of 3 MΩ	700	Cost-effective performance and environmental-friendly structure	Wear of film by friction	[24]
Hosta leaf	Poly(methyl metracrylate) (PMMA)	8 × 8 cm^2^	45 mW·m^−2^ at *R_L_* of 10 MΩ	230 V	Environmental-friendly structure	Fragile structure and without portable frame structure.	[25]
Peanut shell powder	PET	4.5 × 4.5 cm^2^	577 mW·m^−2^ at *R_L_* of 5 MΩ	390 V	Bio-waste and non-toxic material used as a triboelectric layer	Decreased device performance due to the hydrophilic nature of the peanut shell powder	[26]
Rhododendron leaves	Ecoflex pad	5 × 5 cm^2^	~150 mW·m^−2^ at *R_L_* of 200–300 MΩ	140 V	Simple structure and easy working principle	Fragile structure and without portable frame structure.	[27]
Diatom frustule chitosan	FEP	3 × 4 cm^2^	15.7 mW·m^−2^ at *R_L_* of 5 MΩ	150 V	Simple structure and low-cost manufacturing	Stability performance tests are required	[28]
Alginate film	Aluminum	5 × 5 cm^2^	3.8 mW·m^−2^ at *R_L_* of 20 MΩ	33 V	Low toxicity, good biocompatibility, and biodegradability triboelectric film	Fragile structure and without portable frame structure.	[29]
Laver (Korean seaweed)	FEP	2 × 2 cm^2^	2 mW·m^−2^ at *R_L_* of 500 MΩ	-	Flexible, lightweight, and edible structure	Wear of film by friction and without portable frame structure.	[30]
Rumex *vesicarius* leaves powder	Poly(ethylene terephthalate)(PET)/Polytetrafluoroethylene (PTFE) film	5 × 5 cm^2^	1.894 mW·m^−2^ at *R_L_* of 20 MΩ	3.86 V	Simple structure, low-cost fabrication, and stable output performance	Fragile structure	[31]
Dehydrated nopal powder	Polyimide film	5.22 × 5.22 cm^2^	0.556 mW·m^−2^ at *R_L_* of 76.89 MΩ ^a^ 2.309 mW·m^−2^ at *R_L_* of 76.89 MΩ ^b^	16.4 V 38 V	Potable and simple structure, low-cost fabrication, and stable performance	Wear of film by friction	This work

-- * Data not available. ^a^ Test using the shaker. ^b^ Test by applying the hand force.

## Data Availability

Not applicable.

## Supplementary Materials

The following supporting information can be downloaded at: https://www.mdpi.com/article/10.3390/s23094195/s1, Video S1: The output voltage of TENG with NOP and without NOP, Video S2: The output voltage of the NOP-TENG by applying force with the hand, Video S3: TENG powering a digital calculator, Video S4: Lighting of 18 LEDs with the output power of the TENG. Figure S1: Setup of the equipment used to characterize the NOP-TENG.
