# Supplementary material for: A Portable Triboelectric Nanogenerator Based on Dehydrated Nopal Powder for Powering Electronic Devices
Source: Sensors (Basel). 2023 Apr 22;23(9):4195. doi: 10.3390/s23094195 (PMC10180813; doi:10.3390/s23094195)
Supplement: Supplementary file 1 [file sensors-23-04195-s001.zip › sensors-2328396-Supplementary Information.pdf]

Supplementary Information

A Portable Triboelectric Nanogenerator Based on Dehydrated Nopal Powder for Powering Electronic Devices

Ernesto A. Elvira-Hernández [1,2], Omar I. Nava-Galindo [3], Elisa K. Martínez-Lara [4], Enrique Delgado-Alvarado [4,5], Francisco López-Huerta [6], Arxel de León [7], Carlos Gallardo-Vega [8], Agustín L. Herrera-May [4,9,*]

[1] Facultad de Ingeniería Mecánica y Ciencias Navales, Universidad Veracruzana, Calzada Ruiz Cortines 455, Boca del Río 94294, Veracruz, Mexico; aelvirah@hotmail.com
[2] Campus Torrente, Universidad Cristobal Colón, Av. Salvador Díaz Mirón 2602, Veracruz 91910, Veracruz, Mexico
[3] Departamento de Ingeniería Mecánica, DICIS, Universidad de Guanajuato, Salamanca 36885, Guanajuato, Mexico; iem.nava@gmail.com
[4] Micro and Nanotechnology Research Center, Universidad Veracruzana, Calzada Ruiz Cortines 455, Boca del Río 94294, Veracruz, Mexico; karina.24_lara@outlook.es (E.K.M.-L.); endelgado@uv.mx (E.D.-A.)
[5] Facultad de Ciencias Químicas, Universidad Veracruzana, Calzada Ruiz Cortines 455, Boca del Río 94294, Veracruz, Mexico
[6] Facultad de Ingeniería Eléctrica y Electrónica, Universidad Veracruzana, Calzada Ruiz Cortines 455, Boca del Río 94294, Veracruz, Mexico; frlopez@uv.mx
[7] CONACYT-Centro de Investigación en Química Aplicada, Boulevard Enrique Reyna 140, Saltillo 25294, Coahuila, Mexico; arxel.deleon@ciqa.edu.mx
[8] Centro de Investigación en Química Aplicada, Boulevard Enrique Reyna 140, Saltillo 25294, Coahuila, Mexico; carlos.gallardo@ciqa.edu.mx
[9] Facultad de Ingeniería de la Construcción y el Hábitat, Universidad Veracruzana, Calzada Ruíz Cortines 455, Boca del Río 94294, Veracruz, Mexico; leherrera@uv.mx
* Correspondence: leherrera@uv.mx; Tel.: +52-2297752000

Supplementary Video S1

This video shows the characterization of two nanogenerators with and without nopal powder. For this characterization, the parallel electrical connection of the two nanogenerators was made (Figure 6a) and an external force was applied manually. At the same time, the open circuit voltage was recorded through an oscilloscope with a 100 MΩ impedance probe. In video S1, the nanogenerator with dehydrated nopal powder generates a higher peak-to-peak open-circuit voltage (14.56 V) compared to one without dehydrated nopal powder (9.92 V). The result of the voltage of both generators is observed in Figure 6b.

Supplementary Video S2

This video depicts the mechanical characterization performed on the NOP-TENG with a load resistance of 76.89 MΩ connected between its upper and lower electrodes (Figure 8a). For

this characterization, the external force was applied by hand and a maximum closed-circuit voltage was measured on the oscilloscope with a 100 MΩ impedance probe (Figure 8b). This voltage was used to determine the power (6.29 μW) employing the equation $P = V_{max}^2 / R_L$. In addition, a power density of 2309.98 μW·m^{-2} was calculated considering an area of 52.2 mm × 52.2 mm.

Supplementary Video S3

This video demonstrates that the NOP-TENG can power a digital calculator by charging a 22 μF capacitor for 900 seconds. In this experiment, the setup of Figure S1 and shaker vibrations of 15 Hz with 4*g* acceleration were used. Figure 9c shows the connection diagram of the NOP-TENG with the energy storage circuit and the digital calculator. In addition, the voltage on the capacitor during its charge and subsequent discharge when the calculator is turned on is shown (Figure 9d).

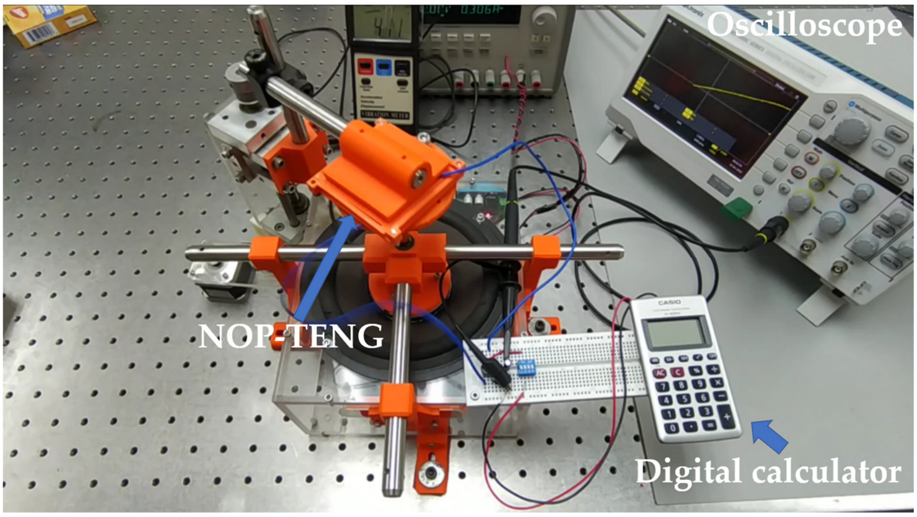

Figure S1. Setup of the equipment used to characterize the NOP-TENG.

Supplementary Video S4

This video shows 18 commercial green LEDs powered by the NOP-TENG (Figure 9e). For this, the nanogenerator was under mechanical vibrations of the shaker to a frequency of 15 Hz with an acceleration of 4*g* and following the diagram of Figure 9e.
