# Supplementary figures and images for: A Portable Triboelectric Nanogenerator Based on Dehydrated Nopal Powder for Powering Electronic Devices
Source: Sensors (Basel). 2023 Apr 22;23(9):4195. doi: 10.3390/s23094195 (PMC10180813; doi:10.3390/s23094195)

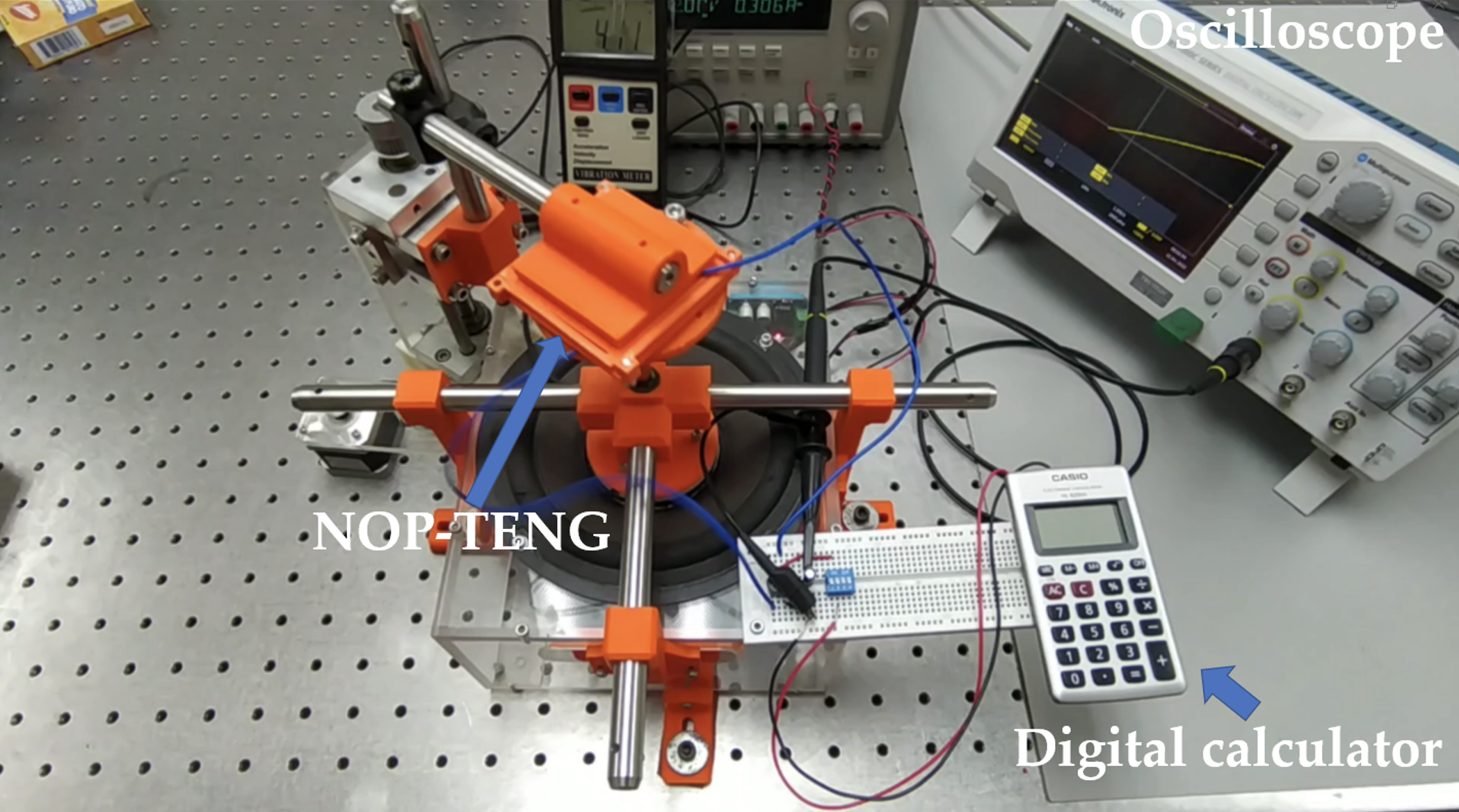

Supplement: Supplementary file 1 [file sensors-23-04195-s001.zip › Figure S1 updated.tiff]
